# A Stochastic Frontier Approach to Study the Relationship between the Hygienic Quality of Bulk Tank Sheep Milk and Technical Efficiency of the Coagulation Process

**DOI:** 10.3390/foods13060873

**Published:** 2024-03-13

**Authors:** Lorena Jiménez, José M. Perea, Javier Caballero-Villalobos, Elena Angón, Alessio Cecchinato, Nicolò Amalfitano, Bonastre Oliete, Ramón Arias

**Affiliations:** 1 Instituto Regional de Investigación y Desarrollo Agroalimentario y Forestal de Castilla La Mancha (IRIAF), CERSYRA de Valdepeñas, 13300 Ciudad Real, Spain; ljimenez@quesomanchego.es (L.J.); rarias@jccm.es (R.A.); 2 Departamento de Producción Animal, Universidad de Córdoba, 14071 Córdoba, Spain; jmperea@uco.es (J.M.P.); eangon@uco.es (E.A.); 3 Department of Agronomy, Food, Natural Resources, Animals and Environment (DAFNAE), University of Padova, Viale dell’Università 16, 35020 Legnaro, Padova, Italy; alessio.cecchinato@unipd.it (A.C.); nicolo.amalfitano@unipd.it (N.A.); 4 Institut Agro, Université Bourgogne Franche-Comté, PAM UMR A 02.102, 21000 Dijon, France; bonastre.oliete@u-bourgogne.fr

**Keywords:** coagulation efficiency, stochastic frontier analysis (SFA), Manchego cheese, dairy industry

## Abstract

Sheep milk from local breeds is important for the production of high-quality cheeses throughout the Mediterranean region, such as Manchego cheese in Spain. To maintain sustainable and efficient production, it is necessary to reach a better understanding of how the composition and hygiene of the milk affect the coagulation process, with the aim of optimizing production yield. This study implemented a stochastic production frontier function to estimate the potential production of curd and efficiency using data from the four seasons of a study of 77 Manchega sheep farms. The Cobb–Douglas production frontier model was estimated using the maximum likelihood estimation method. The results showed that the content of protein, lactose, and fat exhibited increasing returns to scale, with protein content being the most significant factor for curd production. Approximately half of the inefficiency was due to factors related to the technological properties and the hygiene of the milk. The pH, curd firmness, and concentration of lactic acid bacteria improved the efficiency of coagulation, while the concentration of spores of lactate-fermenting *Clostridium* spp., *Pseudomonas* spp., staphylococci, and catalase-negative gram-positive cocci favored the inefficiency of the coagulation process. To date, this is the first study to evaluate the effect of different factors, such as microbial groups, milk composition, and technological properties, on the efficiency of the coagulation process in dairy sheep.

## 1. Introduction

In Mediterranean regions, the production of traditional cheeses using milk from local sheep and goat populations plays a significant role in the economy and supporting communities in disadvantaged rural areas [1,2]. The region of Castilla-La Mancha (Spain) is noteworthy, and is renowned for Manchego cheese, a product covered under a Protected Designation of Origin (PDO), made exclusively with milk from Manchega, probably the most important Spanish native dairy sheep breed [3]. This specific production supports over half a million sheep on 538 farms and aids 72 cheese factories, annually yielding around 17 million kilograms of Manchego cheese [4]. Considering these figures and values of production, it is essential to understand and control milk transformation into cheese, as impairments in this process can severely compromise the economic sustainability of dairy farms [5].

Milk coagulation is a complex transformation process where curd is obtained primarily from fat and protein as inputs. The interaction of biochemical and physical factors during the coagulation process is decisive in the curd consolidation, establishing the beginning of a series of events that define the curd quality and yield [6]. Optimizing this process also minimizes raw material waste in the whey, thereby contributing to the sustainability of cheese production [7]. To achieve an efficient yield, it is critical to understand and manage critical basic points such as the milk composition, hygienic conditions of the process, and technological factors like coagulation time and curd firmness.

The hygienic and sanitary quality of milk can significantly impact the coagulation process and, consequently, the organoleptic properties of cheese and its final yield [8,9,10]. Milk with high somatic cell counts—an indicator of mammary infection—tends to coagulate slower and often leads to lower curd yields [11,12]. Furthermore, the microbiological profile of milk is affected by both its native microbiota and other external sources of post-milking contamination, such as environmental conditions at the farm or cheesemaking facilities [13]. These sources comprise various microorganisms, including yeasts, molds, and bacteria. The influence of these microorganisms on the coagulation process of cow milk has been underexplored, and even less is known about their impact on milk from small ruminants [10]. Overall, these studies show a partial vision of milk coagulation, since only a small number of factors are studied together.

The inherently complex process of milk coagulation could be effectively analyzed and optimized through modeling and analysis within stochastic frontier production functions [14,15]. This approach is of interest to the dairy industry, as it facilitates the assessment of the impacts of various factors, such as microbial groups, milk composition, and its technological properties, on the efficiency of the coagulation process. The inclusion of these elements into the analytical framework of stochastic frontier production functions allows for the identification of determinants of efficiency and the optimal conditions to maximize yield, enabling precise adjustments for more efficient and sustainable production of cheese and other derived dairy products [16,17].

Previous studies have used deterministic parametric methods to assess process efficiency, including coagulation [18,19]. However, the introduction of stochastic frontier analysis (SFA) has led to a more nuanced approach [20]. SFA separates deterministic efficiency from stochastic noise, providing deeper insights into process inefficiencies by considering the inherent variability in production. This approach, therefore, offers a more comprehensive understanding of efficiency.

The primary goal of this study was to utilize SFA for modeling and assessing the technical efficiency of the coagulation process of bulk tank milk from Manchega sheep. Considering that bulk tank milk reflects the quality and composition of milk in a cheese-making industrial context, the findings of this study are expected to provide a comprehensive and representative analysis of the actual production conditions. In addition, the present study also aims to quantify the relative contributions of the principal inputs to the coagulation process and to examine the impacts of key technological properties and milk microbial loads on the inefficiency of the coagulation process, which is an important advanced innovation that could be applied in the Manchego cheese industry.

## 2. Materials and Methods

### 2.1. Dataset and Sample Collection

This study involved 77 Manchega sheep flocks located in the region of Castilla-La Mancha, Spain. These flocks currently comprise approximately 15% of the farms registered under the PDO “Manchego cheese” [4]. Each of the studied flocks consisted of a single breed and operated under a semi-extensive production system associated with grazing on natural pastures, residues, and cereal crop remains. The flock sizes varied, ranging from 150 to 5500 ewes with an average production of 180 kg in 150 days, all of which were mechanically milked. A full description of the farming system can be found in Rivas et al. [21].

Bulk tank samples were collected from each flock at 4 time points (once each season), making a total of 308 samples. These samples were obtained from the tank, composed of a blend of milk from the morning milking of the sampling day and the evening milking of the previous day. The samples were collected into clearly labeled sterile containers, and transported at 4 °C to the Laboratory of Lactology at the Regional Center for Animal Selection and Reproduction (CERSYRA-IRIAF, Valdepeñas, Ciudad Real, Spain) within a maximum timeframe of 2 h. At arrival, the samples were aliquoted and prepared for compositional analysis, microbiological studies, and coagulation tests, all conducted no longer than 48 h after sample collection.

### 2.2. Laboratory Analysis

A first 50 mL aliquot of each milk sample was sent to the Small Ruminant Dairy Laboratory at the University of Córdoba (Córdoba, Spain) for an analysis of the milk coagulation properties (MCP), which was performed within 24 h after sampling. Rennet clothing time (RCT, min), curd firmness at 60 min (A_60_, mm), and curd yield (CY, g/100 mL) were determined at 32 °C using a Formagraph lactodinamograph (Foss Electric A/S, Hillerød, Denmark) [18].

A second 50 mL aliquot of milk had azidiol added and was sent to the Interprofessional Dairy Laboratory of Castilla-La Mancha (LILCAM, Talavera de la Reina, Spain) for a milk composition analysis and somatic cell count (SCC), which were carried out within 48 h after sampling. SCC was subsequently expressed as somatic cell scores (SCS) to normalize its distribution by applying a logarithmic transformation [22]. Fat, protein, and lactose contents were determined using a Milkoscan 6000 FT device (Foss Electric, Hillerød, Denmark). SCC was obtained with a Fossomatic FC (Foss Electric, Hillerød, Denmark), and pH was measured using a Crison Basic20 pHmeter (Crison Instruments, Barcelona, Spain).

A third 50 mL aliquot was used for microbiological analyses at CERSYRA-IRIAF, which were performed within 24 h after sample collection. From each sample, serial dilutions were made to inoculate 0.1 mL onto different culture media. Bacterial counts for the following groups of microorganisms were determined on PCA media (Panreac, Barcelona, Spain): standard plate count (SPC) was incubated in aerobic conditions at 30 °C for 72 h; thermoduric bacteria (THERMO) were incubated in the same conditions as SPC, after pasteurizing milk at 62.8 °C for 30 min; and psychrotrophic bacteria (PSYCHRO) were incubated at 6.5 °C for 10 days. *Pseudomonas* spp. (PSEUDO) were cultured on Cetrimide agar (Panreac, Spain) and incubated at 35 °C for 48 h. The determination of coliforms (COLI) was conducted using CromoIDTM Coli medium (bioMérieux, Madrid, Spain), incubated at 37 °C for 24 h. Gram-positive catalase-negative cocci count (GPCNC) was determined in modified Edwards medium with colistin and oxolinic acid supplement (Oxoid, Basingstoke, UK), incubated at 35 °C for 48 h. Lactic acid bacteria (LAB) were seeded on MRS medium (Panreac, Spain) acidified to pH 5.7, and incubated at 30 °C for 72 h. Lactate-fermenting *Clostridium* spores (LFCS) count was performed using the most probable number (MPN) technique, in Bryant and Burkey Broth (BBB, Merck, Germany). For the enumeration of coagulase-positive staphylococci (CPS) and coagulase-negative staphylococci (CNS), Baird Parker RPF Agar medium (bioMérieux, Spain) was used, and incubated at 37 °C for 24 h. A full description of the microbiological analysis can be found in a previous study from our research group [10]. The microbial counts were subsequently subjected to a decimal logarithmic transformation to normalize their distribution [10].

### 2.3. Stochastic Frontier Analysis of the Milk Coagulation Process

#### 2.3.1. Theoretical Concept

In 1977, several authors [20,23] formulated a stochastic frontier production function that can be specified for panel data as [24]:

(1) $$Y_{it}=e^{X_{it}\beta +V_{it}-U_{it}}$$

where *Y_it_* denotes the output of the *i-th* unit (*i* = 1, *…*, *N*) in the *t-th* time period (*t* = 1, *…*, *T*), *X_it_* is a *k* × 1 vector of input quantities used by the *i-th* unit in the *t-th* time period, *β* is a *k* × 1 vector of coefficients to be estimated, and *V_it_* and *U_it_* are components of the error term assumed to be independent. The first component, *V_it_*, is a normally distributed random variable with a zero mean and variance $\sigma_{v}^{2}$, accounting for measurement errors and other random factors. The second component, *U_it_*, is a non-negative random variable that measures the deviation from the efficient frontier for the *i-th* observation, derived from the normal distribution truncated at zero, with a mean *Z_it_δ* and variance σ^2^. *Z_it_* is a 1 × *m* vector of explanatory variables associated with technical inefficiency over time, and *δ* is an *m* × 1 vector of unknown coefficients [25].

The formulation for inefficiency effects in the panel data model [24] allows for the joint estimation of the stochastic production function, and the model for inefficiency effects linked to this function is presented as follows:

(2) $$U_{it}=Z_{it}\delta +W_{it}$$

where *U_it_* represents the estimated one-sided inefficiency of unit *i* in time period *t*, Z*_it_* is the set of variables explaining the inefficiency of unit *i* in period *t*, *δ* is a set of coefficients estimated in the inefficiency model, and *W_it_* is defined by the truncation of the normal distribution with a mean zero and variance *σ*^2^.

The stochastic production function (1) and the inefficiency effects model (2) are estimated simultaneously using the maximum likelihood method. The technical efficiency (TE) estimates for unit *i* in time period *t* are presented as follows:

(3) $${TE}_{it}=\frac{E\left(Y_{it}^{*}/u_{it},{\;X}_{it}\right)}{E\left(Y_{it}^{*}/u_{it}=0,\;\;X_{it}\right)}=e^{-u_{it}}=e^{-z_{it}\delta -w_{it}}$$

where $Y_{it}^{*}$ is the production, which is equal to $e^{Y_{it}}$ when expressed in logarithms. Therefore, TE is calculated as the ratio of the level of production obtained with respect to the maximum achievable production given the quantities of the inputs (i.e., when *u_it_* = 0). The value of TE ranges from 0 to 1, with the latter being the most favorable.

#### 2.3.2. Empirical Model

The empirical analysis was based on the estimation of a Cobb–Douglas production function, in which both production and inputs are expressed in logarithmic form. Therefore, the estimated coefficients reflect the production elasticities [26]. A translog function was also estimated, whose preliminary results led to the rejection of the functional form [27,28,29].

Each of the 77 Manchega sheep farms was considered as a production unit, and each of the seasons (spring, summer, autumn, and winter) as a time period. The model assumes that the production of curd (CY, g/100 mL) is a function of three inputs, fat (FAT, g/100 mL), protein (CP, g/100 mL), and lactose (LAC, g/100 mL), expressed as:

(4) $${LnY}_{it}=\beta_{0}+\sum_{k=i}^{3}\beta_{k}{LnX}_{kit}+V_{it}-U_{it}$$

where *Y_it_* is the total curd production from a milk sample of farm *i* in season *t*, *X*_1–3_ are the three previously defined variable inputs of farm *i* in season *t*, *V_it_* is the random noise, and *U_it_* is the inefficiency term.

It is assumed that inefficiency follows a half-normal distribution, with the inefficiency model being specified as:

(5) $$U_{it}=\delta_{0}+\sum_{k=1}^{14}\delta_{k}Z_{kit}$$

where *Z* is the explanatory variables (Table 1) and *δ* is a set of parameters to be estimated. The estimation of Equations (5) and (6) is carried out simultaneously by maximum likelihood [24] using the program FRONTIER 4.1. [30].

The existence of inefficiency (H_0_: γ = δ_0_ = δ_1_ = … = δ_14_ = 0), the relevance of exogenous variables in explaining the inefficiency component (H_0_: δ_0_ = δ_1_ = … = δ_14_ = 0), the existence of stochastic efficiency (H_0_: γ = 0), and the assumption of the truncated normal distribution of the inefficiency component (H_0_: *μ* = 0) were assessed using the generalized likelihood ratio statistic (λ), defined as [27,28,29]:

(6) $$\lambda =-2\left\{ln\left[L\left(H_{0}\right)\right]/ln\left[L\left(H_{1}\right)\right]\right\}$$

where ln[(H_0_)] corresponds to the value of the log-likelihood function for the restricted model (specified in the null hypothesis) and ln[(H1)] is the value of the log-likelihood function for the general model stipulated in the alternative hypothesis. This test is asymptotically distributed as a chi-squared distribution with degrees of freedom equal to the difference in the number of parameters estimated under both hypotheses [30].

Finally, the milk samples were classified according to the TE percentile into three groups: low (<P_15_), medium (P_15_ to P_85_), and high (>P_85_). The three efficiency groups were compared using simple ANOVA and the SNK test. Additionally, the association between TE and the season of the year was analyzed using the same methods (ANOVA and SNK test). Statistical analyses were performed using the software XLSTAT v.19.4 [31].

## 3. Results and Discussion

Table 2 presents the results from the estimation of the stochastic frontier production function for the coagulation of Manchega sheep milk, using a Cobb–Douglas production model. All obtained β coefficients were different from zero (*p* < 0.05). The highest output elasticity was for protein, at 0.94, implying that a 1% increase in milk protein content would increase curd production by 0.94%. The lowest output elasticity corresponded to lactose content, while fat content had an elasticity of 0.46.

The elasticity of milk components is influenced by a range of factors, both environmental (e.g., diet) and intrinsic (e.g., genetics of the breed). Fat content in milk can be adjusted through dietary modifications, but milk protein shows a more pronounced genetic dependence [32,33]. Considering the significant role of protein in enhancing the efficiency of coagulation, prioritizing its improvement through genetic selection programs is deemed essential. The necessity for such targeted improvements is underscored by the lack of research in this area, with only a few studies addressing this topic [19]. Furthermore, expanding this line of research to include other breeds, species, and production systems would be valuable, as it would provide a more comprehensive understanding of the efficiency of the coagulation process, encompassing both technical and economic aspects.

The sum of all output elasticities was 1.83, indicating that, on average, the Manchega dairy sheep system has increasing returns to scale. This means that if the fat, protein, and lactose contents in Manchega sheep milk were to increase by 1%, there would be a 1.83% rise in curd yield, leading to financial benefits for the industry. It is important to consider the negative correlation that usually exists between lactose content and fat and protein content, due to the role of lactose in the regulation of milk volume, which can lead to a dilution effect of the other major milk components. This fact could pose a challenge to achieving an effective increase in scale performance [34]. From a practical perspective, despite this negative correlation, an effective strategy could be a combination of milks with different fat, protein, and lactose contents to try to achieve an optimal balance by taking advantage of variations in population. An assessment of the technical and economic feasibility of this approach would be of interest.

On the other hand, milk quality payment systems often equally value fat and protein content, considering the sum of both (“cheese extract”) or total solids. However, the findings of this study suggest that protein content has a greater importance than fat content regarding curd yield in Manchega sheep. Therefore, it is important to conduct a more detailed economic assessment to adapt these discoveries to the pricing system. Additionally, it should be noted that this study did not take into account the composition of the protein, meaning that the impact of casein was not distinguished from that of whey proteins. Such an adjustment would allow for a fairer and more accurate reward for producers, based on the true cheesemaking yield of the supplied milk. This reassessment is essential not only for reasons of social equity, but also to enhance the economic efficiency of the dairy sector. By better aligning financial rewards with factors that genuinely improve the quality and yield of cheese production, producers would be incentivized to optimize milk quality, thus promoting continuous improvement in the sector [35]. This more exhaustive and quality-based approach could encourage innovations in farm management and feeding practices, which, in turn, could lead to more sustainable and profitable long-term dairy production.

**Figure 1:**
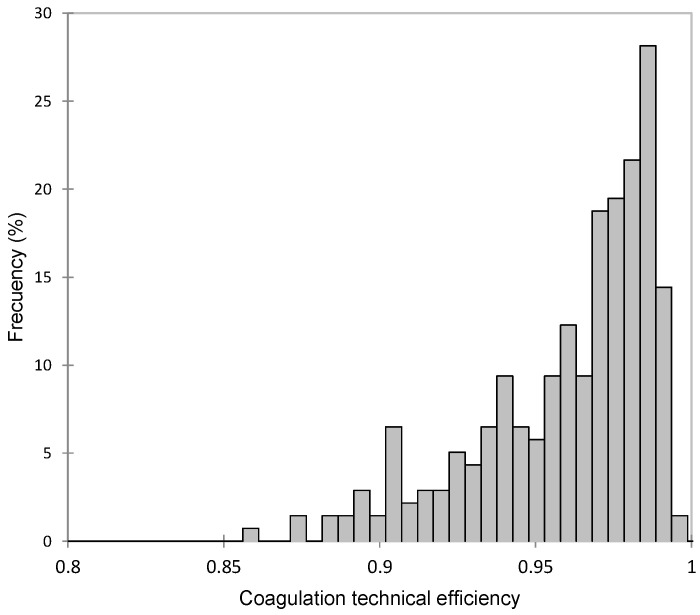
Frequency distribution of coagulation technical efficiency (TE) in Manchega sheep.

The average TE for coagulation was 0.95, ranging from 0.86 to 0.99 (Figure 1). Therefore, the average inefficiency in curd production was 0.05 (1-TE), which translates to a mean marginal loss of 52.6 g of curd per kilogram of curd produced, according to the average composition shown in Table 1. Previous studies have reported lower TE values, although their results are not entirely comparable, as they modeled a Cobb–Douglas function with two predictors (fat and protein content) using ordinary least squares (OLS) and assessed efficiency through a deterministic frontier [19].

The tests on the specifications of the model for technical inefficiency led to the rejection of all null hypotheses under consideration (*p* < 0.05). Therefore, it is confirmed that an average production function constitutes an inadequate representation of the data, the necessity to incorporate technical inefficiency in the production function, the significance of the variables that explain technical inefficiency, and the fit to the truncated normal of the inefficiency component.

The variance parameter γ was 0.51, indicating that half of the variation in the error of the function was due to the inefficiency error u_it_, while the other half was due to the stochastic random error ν_it_.

Table 2 presents the technical inefficiencies identified in the model. Positive parameter estimates indicate relative technical inefficiency, while negative ones signify relative technical efficiency. pH and A_60_ were statistically significant in the inefficiency model, suggesting that coagulation efficiency improves with an increased curd firmness and higher pH levels.

Ten groups of microorganisms were considered in the inefficiency model. Of these, six were statistically significant in their effect on the (in)efficiency of the coagulation process: LFCS, LAB, PSEUDO, GPCNC, CPS, and CNS. The concentration of these groups of microorganisms, with the exception of LAB, was associated with an increase in the inefficiency of the coagulation process. In contrast, a higher concentration of lactic acid bacteria (LAB) was associated with a greater efficiency in the process, suggesting a positive effect of this group of microorganisms on coagulation.

The more pronounced the magnitude of efficiency or inefficiency, the greater the deviation of the estimated value from zero. A detailed examination of each parameter estimate reveals noteworthy insights. Notably, pH emerged as the most influential factor in the inefficiency model, showing a positive impact on the efficiency of the process. This relationship is also reflected in Table 3, where three levels of efficiency are differentiated based on the 15th and 85th percentiles. An increase in average pH is observed, going from 6.49 in the least efficient group (15th percentile) to 6.74 in the most efficient group (85th percentile). pH is related to the coagulation process [36]: acidic pHs are associated with quicker coagulations, whereas pHs leaning towards alkaline tend to slow down the process, resulting in firmer curds [37], which, according to our findings, lead to a greater coagulation efficiency due to improved solid retention [38].

Although RCT was not found to be significant in the inefficiency model, Table 3 shows an average increase in both RCT and A_60_ values from the lowest efficiency group to the highest. This indicates that an optimal pH is crucial for improving the TE of milk coagulation by influencing both the physical properties of the curd and its interaction with the microbiota. This approach confirms the idea that, in order to optimize the quality and efficiency of cheese production, it is necessary to consider milk physicochemical and microbiological attributes.

PSEUDO and CNS were the bacterial groups with the most negative impact on the milk coagulation process (Table 2). CNS is a group that includes a range of microorganisms that typically cause subclinical intramammary infections in sheep, characterized by moderately elevated but persistent cell counts, mainly affecting animals with high productivity [39]. Such infections can lead to minor changes in milk composition, like alterations in protein levels and somatic cell count, which can negatively influence curd quality and yield [10,40].

PSEUDO are ubiquitous microorganisms capable of surviving and proliferating at low temperatures, often associated with poor hygiene conditions, and spread during extended periods of milk storage [41,42]. Specifically, the presence of enzymes such as proteases and lipases, produced by some members of this group, is particularly problematic for cheesemaking. These enzymes break down the fat and protein in milk, potentially altering the structure and integrity of the curd. This not only affects the texture and quality of cheese, but can also reduce the curd yield due to a lower retention of solids and essential nutrients [10,43]. Although the PSYCHRO group was not significantly relevant in the inefficiency model, Table 3 indicates a decrease in the average concentration of these microorganisms when moving from low to high efficiency groups.

The GPCNC group exhibited an adverse effect on the efficiency of the coagulation process, though its impact was moderate compared to other microbial groups. This group includes various bacterial species that are indicators of poor hygiene conditions in the milk production environment, as well as mammary health issues in the breed [13,44,45]. From a coagulation perspective, these microorganisms can negatively impact the process by altering the concentration of minerals, the balance of proteins and fats, and enzymatic activity, which, in turn, can influence the formation and texture of the curd [10,46].

LFCS also showed a moderate negative effect on the efficiency of Manchega sheep milk coagulation. Furthermore, these microorganisms cause late blowing in pressed cheese, leading to the formation of cracks and cavities due to acid-butyric fermentation by vegetative cells once the sporulated forms germinate inside the cheese, causing significant economic losses for the cheese industry. These microorganisms are primarily introduced into the milk through silage and other by-products used in livestock feed, as well as by poor hygiene in the milking parlor [47]. Additionally, a correlation has been described between high spore counts and the technological characteristics of the milk, with increases in coagulation time and curd firming time [10]. Therefore, the presence of LFCS is a critical factor that must be controlled to ensure quality and efficiency in the production of fermented dairy products.

The CPS group also showed a minor negative impact on the efficiency of the milk coagulation process. *Staphylococcus aureus* is one of the main pathogens causing clinical mastitis in dairy ruminants [48,49]. Its significance extends beyond animal health, also impacting public health, as they are known for producing thermostable toxins [50,51]. From the perspective of milk coagulation, the presence of CPS can interfere with the normal process due to several factors. Firstly, mammary infections caused by these organisms can alter the chemical composition of the milk, affecting its ability to form an adequate curd due to the presence of enzymes from the plasmin–plasminogen complex associated with high somatic cell counts [52,53]. Although their impact on coagulation efficiency is not as marked as other microorganisms, the presence of coagulase-positive staphylococci is an important factor to consider in managing the quality and safety of dairy products. Their control is essential not only for maintaining production efficiency, but also for ensuring the safety of the final product.

On the contrary, LAB were revealed in the inefficiency model as a factor with a significant positive influence on the TE of the coagulation process. This finding is consistent with previous expectations, given the known beneficial role of LAB in lactic fermentation and in the production of dairy products, providing differential organoleptic and sensory characteristics to cheeses [54,55]. In addition, LAB contribute to the inhibition of other undesirable microorganisms present in raw milk [56,57]. Therefore, their presence helps to maximize curd efficiency and cheese production. It is important to consider that expecting both alkaline pH and LAB to improve coagulation performance may seem contradictory. It is crucial to note that the initial pH of milk, ranging between 6.00 and 7.00, according to Table 1, does not show more acidic values indicative of the massive proliferation of LAB. The acidifying capacity of LAB depends on the strain, as well as its homo- or heterofermentative aptitude [58,59]. Moreover, the microbiome of raw sheep milk is highly complex [60], and the behavior of LAB may differ from that of commercial LAB starters, which are designed, among other aspects, to have a high acidifying capacity. It could be of interest to explore whether there are specific conditions under which alkaline pH and LAB could synergistically interact to improve coagulation performance, even within a range of initial milk pH close to neutrality.

**Figure 2:**
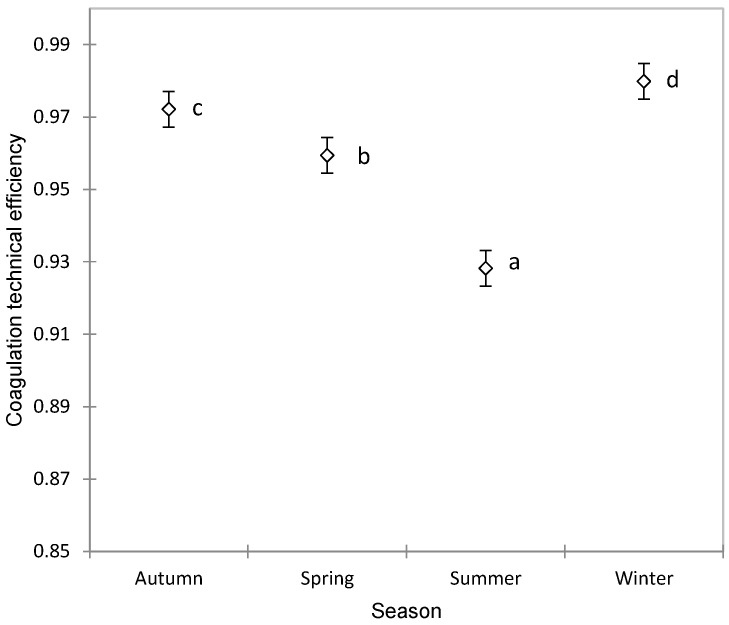
Association between the season and coagulation technical efficiency in Manchega sheep using ANOVA (mean ± standard error). Means without a common superscript (a–d) are statistically different (Student–Newman–Keuls, SNK *p* < 0.05).

The TE showed significant variations depending on the season, increasing in autumn and reaching its highest values in winter, then decreasing in spring and recording the lowest values in summer, as observed in Figure 2. This variation is even more important, because the production of Manchega sheep milk does not have large seasonal fluctuations as occurs in other dairy breeds such as Sarda [61] or Latxa [62]. Therefore, this seasonal variability could well be due to climatic conditions, which selectively affect contamination by different groups of microorganisms [63,64,65] or in the composition of the milk due to aspects related to grazing, types of forage and preserved foods, periods of stabling, ventilation, and other related factors [66].

## 4. Conclusions

This study implements a Cobb–Douglas stochastic production frontier function to estimate the potential production and technical efficiency of curd production from Manchega sheep milk. Using data from 77 farms, this study details, for the first time, the determinants of potential yields for the dairy sheep industry under different production constraints using a stochastic approach. The main findings of this study are: (1) empirical results showing that the Cobb–Douglas stochastic production frontier function model fits the data better than the translog specification; (2) curd production shows an increasing returns to scale, meaning a 1% increase in all input factors would result in almost 2% increase in production; (3) this study estimates substitution elasticities to identify that milk protein content is the most relevant input for curd production; and (4) approximately half of the inefficiency was due to factors related to the technological properties and hygiene of the milk. The pH, curd firmness, and concentration of lactic acid bacteria improve the efficiency of coagulation, while the concentration of spores of lactate-fermenting *Clostridium* spp., pseudomonas, staphylococci, and catalase-negative gram-positive cocci favor the inefficiency of the coagulation process.

## Figures and Tables

**Table 1:** Descriptive statistics for variables in the stochastic frontier production function and variables in the inefficiency equation.

Variable	Symbol	Mean	SD	Minimum	Maximum
CY ^1^	Y	31.52	4.88	20.10	44.70
FAT ^1^	X_1_	7.87	0.82	5.74	10.38
CP ^1^	X_2_	6.05	0.60	5.03	8.05
LAC ^1^	X_3_	4.60	0.24	3.71	5.16
pH	Z_1_	6.61	0.15	6.00	7.00
RCT ^2^	Z_2_	31.25	12.12	4.45	50.10
A_60_^3^	Z_3_	33.24	10.49	1.66	54.90
SCS ^4^	Z_4_	6.40	0.77	4.44	8.90
LFCS ^5^	Z_5_	3.41	0.65	0.00	5.04
LAB ^5^	Z_6_	4.89	0.72	2.91	6.48
THERMO ^5^	Z_7_	3.23	0.87	0.00	5.48
PSYCHRO ^5^	Z_8_	4.51	2.08	0.00	8.00
PSEUDO ^5^	Z_9_	3.12	0.85	0.00	4.53
SPC ^5^	Z_10_	5.65	0.83	0.00	8.01
GPCNC ^5^	Z_11_	4.10	0.93	0.00	5.48
COLI ^5^	Z_12_	3.13	0.98	0.00	5.48
CPS ^5^	Z_13_	2.46	1.41	0.00	5.48
CNS ^5^	Z_14_	4.25	0.71	0.00	5.48

^1^ g/100 mL; ^2^ min.; ^3^ mm; ^4^ SCS = log_2_(SCC/100,000) + 3/mL; ^5^ log_10_CFU/mL; CY = curd yield; FAT = fat; CP = protein; LAC = lactose; A_60_ = curd firmness at 60 min; RCT = rennet clotting time; SCS = bull tank milk somatic cell score; LFCS = lactate-fermenting *Clostridium* spores; LAB = lactic acid bacteria; THERMO = thermodurics, PSYCHRO = psychrotrophs, PSEUDO = *Pseudomonas* spp.; SPC = total mesophilic bacteria (standard plate count); GPCNC = gram-positive catalase-negative cocci; COLI = coliforms other than *Escherichia coli*, CPS = coagulase-positive staphylococci, and CNS = coagulase-negative staphylococci.

**Table 2:** Maximum-likelihood estimates parameters for the stochastic frontier production function and inefficiency function equation.

Variable	Parameter	Coefficient	SE	*t*-Ratio	*p*-Value
Constant	β_0_	0.103	0.176	0.59	0.336
Ln(FAT) ^1^	β_1_	0.463	0.064	7.20	<0.001
Ln(CP) ^1^	β_2_	0.941	0.112	8.39	<0.001
Ln(LAC) ^1^	β_3_	0.429	0.148	2.89	0.006
Inefficiency model					
Constant	*δ*_0_	0.734	0.254	2.88	0.007
pH	δ_1_	−0.128	0.039	−3.27	0.002
RCT ^2^	δ_2_	0.000	0.000	0.33	0.378
A_60_^3^	δ_3_	−0.001	0.001	−2.29	0.029
SCS ^4^	δ_4_	−0.007	0.007	−1.05	0.229
LFCS ^5^	δ_5_	0.009	0.005	2.39	0.023
LAB ^5^	δ_6_	−0.022	0.008	−2.69	0.011
THERMO ^5^	δ_7_	−0.003	0.007	−0.48	0.356
PSYCHRO ^5^	δ_8_	0.001	0.003	0.43	0.363
PSEUDO ^5^	δ_9_	0.025	0.010	2.47	0.020
SPC ^5^	δ_10_	0.011	0.010	1.17	0.200
GPCNC ^5^	δ_11_	0.014	0.008	2.16	0.039
COLI ^5^	δ_12_	−0.003	0.006	−0.48	0.355
CPS ^5^	δ_13_	0.005	0.003	2.11	0.043
CNS ^5^	δ_14_	0.025	0.013	2.22	0.034
Error variance	*σ_e_*	0.002	0.000	5.44	<0.001
Variance	*γ*	0.508	0.167	3.04	0.004
Log-likelihood value	-	492.77			

^1^ g/100 mL; ^2^ min.; ^3^ mm; ^4^ SCS = log_2_(SCC/100,000) + 3/mL; ^5^ log_10_CFU/mL; FAT = fat; CP = protein; LAC = lactose; A_60_ = curd firmness at 60 min.; RCT = rennet clotting time; SCS = bulk tank milk somatic cell score; LFCS = lactate-fermenting *Clostridium* spores; LAB = lactic acid bacteria; THERMO = thermodurics, PSYCHRO = psychrotrophs, PSEUDO = *Pseudomonas* spp.; SPC = total mesophilic bacteria (standard plate count); GPCNC = gram-positive catalase-negative cocci; COLI = coliforms other than *Escherichia coli*, CPS = coagulase-positive staphylococci, and CNS = coagulase-negative staphylococci.

**Table 3:** Comparison between the three coagulation efficiency groups using ANOVA.

Variable	Low (<P_15_)	Medium (P_15_ to P_85_)	High (>P_85_)	SEM	*p*-Value
TE (%)	90.58 ^a^	96.52 ^b^	98.90 ^c^	0.01	<0.001
CY (g/100 mL)	25.62 ^a^	31.84 ^b^	36.25 ^c^	0.29	<0.001
FAT (g/100 mL)	7.72	7.86	7.96	0.05	0.410
CP (g/100 mL)	5.92	6.07	6.16	0.03	0.086
LAC (g/100 mL)	4.65	4.59	4.62	0.01	0.414
pH (−log [H+])	6.49 ^a^	6.60 ^b^	6.74 ^c^	0.01	<0.001
RCT (min)	24.90 ^a^	32.21 ^b^	33.20 ^b^	0.73	0.001
A_60_ (mm)	30.25 ^a^	33.13 ^ab^	35.86 ^b^	0.64	0.035
SCS ^1^	6.37	6.46	6.47	0.05	0.657
LFCS ^2^	3.30	3.38	3.49	0.04	0.422
LAB ^2^	4.41 ^a^	4.87 ^b^	5.47 ^c^	0.04	<0.001
THERMO ^2^	3.51	3.23	3.11	0.05	0.077
PSYCHRO ^2^	5.56 ^b^	4.30 ^a^	4.59 ^a^	0.13	0.002
PSEUDO ^2^	4.02 ^c^	3.13 ^b^	2.22 ^a^	0.05	<0.001
SPC ^2^	5.60 ^a^	5.63 ^a^	5.91 ^b^	0.05	0.138
GPCNC ^2^	4.87 ^c^	4.12 ^b^	3.48 ^a^	0.05	<0.001
COLI ^2^	3.76 ^c^	3.08 ^b^	2.76 ^a^	0.06	<0.001
CPS ^2^	2.81	2.42	2.36	0.08	0.236
CNS ^2^	4.68 ^c^	4.27 ^b^	3.79 ^a^	0.04	<0.001

^1^ SCS = log_2_(cells/100,000) + 3; ^2^ log_10_UFC; TE = technical efficiency; CY = curd yield; FAT = fat; CP = protein; LAC = lactose; A_60_ = curd firmness at 60 min.; RCT = rennet clotting time; SCS = bulk tank milk somatic cell score; LFCS = lactate-fermenting *Clostridium* spores; LAB = lactic acid bacteria; THERMO = thermodurics, PSYCHRO = psychrotrophs, PSEUDO = *Pseudomonas* spp.; SPC = total mesophilic bacteria (standard plate count); GPCNC = gram-positive catalase-negative cocci; COLI = coliforms other than *Escherichia coli*, CPS = coagulase-positive staphylococci, and CNS = coagulase-negative staphylococci. ^a–c^: Means without a common superscript are statistically different.

## Data Availability

The raw data supporting the conclusions of this article will be made available by the authors on request.

## References

[1] Laranjo M., Potes M.E. (2022). Traditional Mediterranean Cheeses: Lactic Acid Bacteria Populations and Functional Traits. Lactic Acid Bacteria in Food Biotechnology.

[2] De Devitiis B., Bimbo F., Viscecchia R., Nardone G., Seccia A., Monacis L., Albenzio M., Santillo A. (2023). Consumer Acceptance for Sheep Milk–Based Yogurt—Evidence from a Large Sample of Italian Consumers. J. Dairy Sci..

[3] Ramos I.M., Rodríguez-Sánchez S., Palop M.L., Poveda J.M. (2024). Reduction in the Biogenic Amine Content of Raw Milk Manchego Cheese by Using Biogenic-Amine-Degrading Lactic Acid Bacteria. Food Control.

[4] PDO Queso Manchego (2023). Activities Report of the PDO “Queso Manchego” (Unpublished Data).

[5] Abdelgawad A.R., Rovai M., Caja G., Leitner G., Castillo M. (2016). Evaluating Coagulation Properties of Milk from Dairy Sheep with Subclinical Intramammary Infection Using near Infrared Light Scatter. A Preliminary Study. J. Food Eng..

[6] Fox P.F., Uniacke-Lowe T., McSweeney P.L.H., O’Mahony J.A. (2015). Dairy Chemistry and Biochemistry.

[7] Stocco G., Summer A., Cipolat-Gotet C., Malacarne M., Cecchinato A., Amalfitano N., Bittante G. (2021). The Mineral Profile Affects the Coagulation Pattern and Cheese-Making Efficiency of Bovine Milk. J. Dairy Sci..

[8] Lucey J.A. (2002). Formation and Physical Properties of Milk Protein Gels. J. Dairy Sci..

[9] Le Maréchal C., Seyffert N., Jardin J., Hernandez D., Jan G., Rault L., Azevedo V., François P., Schrenzel J., van de Guchte M. (2011). Molecular Basis of Virulence in Staphylococcus Aureus Mastitis. PLoS ONE.

[10] Jiménez L., Caballero-Villalobos J., Garzón A., Oliete B., Pérez-Guzmán M.D., Arias R. (2023). Exploring the Relationships between Coagulation, Composition, and Hygienic Quality of Bulk Tank Milk from Manchega Sheep. Small Rumin. Res..

[11] Pazzola M., Cipolat-Gotet C., Bittante G., Cecchinato A., Dettori M.L., Vacca G.M. (2018). Phenotypic and Genetic Relationships between Indicators of the Mammary Gland Health Status and Milk Composition, Coagulation, and Curd Firming in Dairy Sheep. J. Dairy Sci..

[12] Paschino P., Vacca G.M., Dettori M.L., Pazzola M. (2019). An Approach for the Estimation of Somatic Cells’ Effect in Sarda Sheep Milk Based on the Analysis of Milk Traits and Coagulation Properties. Small Rumin. Res..

[13] Jayarao B.M., Pillai S.R., Sawant A.A., Wolfgang D.R., Hegde N.V. (2004). Guidelines for Monitoring Bulk Tank Milk Somatic Cell and Bacterial Counts. J. Dairy Sci..

[14] Van der Voort M., Van Meensel J., Lauwers L., Vercruysse J., Van Huylenbroeck G., Charlier J. (2014). A Stochastic Frontier Approach to Study the Relationship between Gastrointestinal Nematode Infections and Technical Efficiency of Dairy Farms. J. Dairy Sci..

[15] Lampe H.W., Hilgers D. (2015). Trajectories of Efficiency Measurement: A Bibliometric Analysis of DEA and SFA. Eur. J. Oper. Res..

[16] Lawson L.G., Agger J.F., Lund M., Coelli T. (2004). Lameness, Metabolic and Digestive Disorders, and Technical Efficiency in Danish Dairy Herds: A Stochastic Frontier Production Function Approach. Livest. Prod. Sci..

[17] Pérez-Méndez J.A., Roibás D., Wall A. (2020). Somatic Cell Counts, Reproduction Indicators, and Technical Efficiency in Milk Production: A Stochastic Frontier Analysis for Spanish Dairy Farms. J. Dairy Sci..

[18] Caballero-Villalobos J., Perea J.M., Angón E., Arias R., Garzón A. (2018). Coagulation Efficiency and Its Determinant Factors: A Case Study for Manchega Ewe Milk in the Region of Castilla-La Mancha, Spain. J. Dairy Sci..

[19] Garzón A., Perea J.M., Arias R., Angón E., Caballero-Villalobos J. (2023). Efficiency of Manchega Sheep Milk Intended for Cheesemaking and Determination of Factors Causing Inefficiency. Animals.

[20] Aigner D., Lovell C.A.K., Schmidt P. (1977). Formulation and Estimation of Stochastic Frontier Production Function Models. J. Econom..

[21] Rivas J., García A., Toro-Mujica P., Angón E., Perea J., Morantes M., Dios-Palomares R. (2014). Technical, Social and Commercial Profile of the Manchega Dairy Sheep Farms in South-Central Spain. Rev. Mex. Cienc. Pecu..

[22] Ali A.K.A., Shook G.E. (1980). An Optimum Transformation for Somatic Cell Concentration in Milk. J. Dairy Sci..

[23] Meeusen W., van Den Broeck J. (1977). Efficiency Estimation from Cobb-Douglas Production Functions with Composed Error. Int. Econ. Rev..

[24] Battese G.E., Coelli T.J. (1995). A Model for Technical Inefficiency Effects in a Stochastic Frontier Production Function for Panel Data. Empir. Econ..

[25] Coelli T., Rao D.S.P., Battese G.E. (1998). An Introduction to Efficiency Effects in Stochastic Frontier Function for Panel Data. Empir. Econ..

[26] Kumbhakar S.C., Lovell C.A.K. (2000). Stochastic Frontier Analysis.

[27] Chiang F.-S., Sun C.-H., Yu J.-M. (2004). Technical Efficiency Analysis of Milkfish (Chanos Chanos) Production in Taiwan—An Application of the Stochastic Frontier Production Function. Aquaculture.

[28] Zewdie M.C., Moretti M., Tenessa D.B., Ayele Z.A., Nyssen J., Tsegaye E.A., Minale A.S., Van Passel S. (2021). Agricultural Technical Efficiency of Smallholder Farmers in Ethiopia: A Stochastic Frontier Approach. Land.

[29] Guo J., Wang Y., Xu H., Li B., Wang Y. (2023). The Financial Density and Improvement of Urban Technological Efficiency: An Estimation Based on the Stochastic Frontier Approach. Land.

[30] Coelli T. A Guide to FRONTIER Version 4.1: A Computer Program for Stochastic Frontier Production and Cost Function Estimation. https://tarjomefa.com/wp-content/uploads/2017/07/7209-English-TarjomeFa.pdf.

[31] Lumivero XLSTAT Statistical and Data Analysis Solution. https://www.xlstat.com/es.

[32] Pulina G., Nudda A., Battacone G., Cannas A. (2006). Effects of Nutrition on the Contents of Fat, Protein, Somatic Cells, Aromatic Compounds, and Undesirable Substances in Sheep Milk. Anim. Feed. Sci. Technol..

[33] Morand-Fehr P., Fedele V., Decandia M., Le Frileux Y. (2007). Influence of Farming and Feeding Systems on Composition and Quality of Goat and Sheep Milk. Small Rumin. Res..

[34] Pulina G., Macciotta N., Nudda A. (2005). Milk Composition and Feeding in the Italian Dairy Sheep. Ital. J. Anim. Sci..

[35] Raynal-Ljutovac K., Lagriffoul G., Paccard P., Guillet I., Chilliard Y. (2008). Composition of Goat and Sheep Milk Products: An Update. Small Rumin. Res..

[36] Caballero-Villalobos J., Garzón A.I., Oliete B., Arias R., Jiménez L., Núñez N., Martínez A.L. (2015). Relationship of Somatic Cell Count and Composition and Coagulation Properties of Ewe’s Milk. Mljekarstvo.

[37] Bencini R. (2002). Factors Affecting the Clotting Properties of Sheep Milk. J. Sci. Food Agric..

[38] Figueroa Sánchez A., Perea Muñoz J., Caballero-Villalobos J., Arias Sánchez R., Garzón A., Angón Sánchez de Pedro E. (2021). Coagulation Process in Manchega Sheep Milk from Spain: A Path Analysis Approach. J. Dairy Sci..

[39] Gonzalo C., Ariznabarreta A., Carriedo J.A., San Primitivo F. (2002). Mammary Pathogens and Their Relationship to Somatic Cell Count and Milk Yield Losses in Dairy Ewes. J. Dairy Sci..

[40] Leitner G., Rovai M., Merin U. (2019). Clinical and Subclinical Intrammamay Infection Caused by Coagulase Negative Staphylococci Negatively Affect Milk Yield and Its Quality in Dairy Sheep. Small Rumin. Res..

[41] Fagundes C.M., Fischer V., da Silva W.P., Carbonera N., Araújo M.R. (2006). Presença de Pseudomonas Spp Em Função de Diferentes Etapas Da Ordenha Com Distintos Manejos Higiênicos e No Leite Refrigerado. Ciência Rural.

[42] De Jonghe V., Coorevits A., Van Hoorde K., Messens W., Van Landschoot A., De Vos P., Heyndrickx M. (2011). Influence of Storage Conditions on the Growth of *Pseudomonas* Species in Refrigerated Raw Milk. Appl. Environ. Microbiol..

[43] Bruzaroski S.R., Correia S.d.S., Devara L.F.d.S., Poli-Frederico R.C., Fagnani R., de Santana E.H.W. (2023). Influence of the Storage Temperature of Raw Sheep Milk on the Spoilage Potential of *Pseudomonas* spp.. Small Rumin. Res..

[44] Marogna G., Rolesu S., Lollai S., Tola S., Leori G. (2010). Clinical Findings in Sheep Farms Affected by Recurrent Bacterial Mastitis. Small Rumin. Res..

[45] De Garnica M.L., Linage B., Carriedo J.A., De La Fuente L.F., García-Jimeno M.C., Santos J.A., Gonzalo C. (2013). Relationship among Specific Bacterial Counts and Total Bacterial and Somatic Cell Counts and Factors Influencing Their Variation in Ovine Bulk Tank Milk. J. Dairy Sci..

[46] Franz C. (2003). Enterococci in Foods—A Conundrum for Food Safety. Int. J. Food Microbiol..

[47] Arias C., Oliete B., Seseña S., Jimenez L., Pérez-Guzmán M.D., Arias R. (2013). Importance of On-Farm Management Practices on Lactate-Fermenting *Clostridium* spp. Spore Contamination of Manchega Ewe Milk: Determination of Risk Factors and Characterization of Clostridium Population. Small Rumin. Res..

[48] Menzies P.I., Ramanoon S.Z. (2001). Mastitis of Sheep and Goats. Vet. Clin. N. Am. Food Anim. Pract..

[49] Gonzalo C., Tardáguila J.A., De La Fuente L.F., San Primitivo F. (2004). Effects of Selective and Complete Dry Therapy on Prevalence of Intramammary Infection and on Milk Yield in the Subsequent Lactation in Dairy Ewes. J. Dairy Res..

[50] Poli A., Guglielmini E., Sembeni S., Spiazzi M., Dellaglio F., Rossi F., Torriani S. (2007). Detection of Staphylococcus Aureus and Enterotoxin Genotype Diversity in Monte Veronese, a Protected Designation of Origin Italian Cheese. Lett. Appl. Microbiol..

[51] Little C.L., Rhoades J.R., Sagoo S.K., Harris J., Greenwood M., Mithani V., Grant K., McLauchlin J. (2008). Microbiological Quality of Retail Cheeses Made from Raw, Thermized or Pasteurized Milk in the UK. Food Microbiol..

[52] Charismiadou M., Karla G., Theodorou G., Goliomytis M., Politis I. (2015). The Effect of Health Status of the Udder on Plasminogen Activator Activity of Milk Somatic Cells in Ovine Milk. Small Rumin. Res..

[53] Caballero-Villalobos J., Garzón A.I., Martínez Marín A.L., Arias R., Ciocia F., McSweeney P.L.H. (2018). Plasmin Activity in Manchega Ewe Milk: The Effect of Lactation, Parity and Health of the Udder, and Its Influence on Milk Composition and Rennet Coagulation. Small Rumin. Res..

[54] Poveda J.M., Cabezas L., McSweeney P.L.H. (2004). Free Amino Acid Content of Manchego Cheese Manufactured with Different Starter Cultures and Changes throughout Ripening. Food Chem..

[55] Smit G., Smit B.A., Engels W.J.M. (2005). Flavour Formation by Lactic Acid Bacteria and Biochemical Flavour Profiling of Cheese Products. FEMS Microbiol. Rev..

[56] Bouchard D.S., Seridan B., Saraoui T., Rault L., Germon P., Gonzalez-Moreno C., Nader-Macias F.M.E., Baud D., François P., Chuat V. (2015). Lactic Acid Bacteria Isolated from Bovine Mammary Microbiota: Potential Allies against Bovine Mastitis. PLoS ONE.

[57] Rodríguez-Sánchez S., Ramos I.M., Rodríguez-Pérez M., Poveda J.M., Seseña S., Palop M.L. (2022). Lactic Acid Bacteria as Biocontrol Agents to Reduce Staphylococcus Aureus Growth, Enterotoxin Production and Virulence Gene Expression. LWT.

[58] Widyastuti Y., Rohmatussolihat, Febrisiantosa A. (2014). The Role of Lactic Acid Bacteria in Milk Fermentation. Food Nutr. Sci..

[59] Toquet M., Gómez-Martín Á., Bataller E. (2021). Review of the Bacterial Composition of Healthy Milk, Mastitis Milk and Colostrum in Small Ruminants. Res. Vet. Sci..

[60] Castro I., Alba C., Aparicio M., Arroyo R., Jiménez L., Fernández L., Arias R., Rodríguez J.M. (2019). Metataxonomic and Immunological Analysis of Milk from Ewes with or without a History of Mastitis. J. Dairy Sci..

[61] Manca M.G., Serdino J., Gaspa G., Urgeghe P., Ibba I., Contu M., Fresi P., Macciotta N.P.P. (2016). Derivation of Multivariate Indices of Milk Composition, Coagulation Properties, and Individual Cheese Yield in Dairy Sheep. J. Dairy Sci..

[62] Abilleira E., Virto M., Nájera A.I., Salmerón J., Albisu M., Pérez-Elortondo F.J., Ruiz de Gordoa J.C., de Renobales M., Barron L.J.R. (2010). Effects of Seasonal Changes in Feeding Management under Part-Time Grazing on the Evolution of the Composition and Coagulation Properties of Raw Milk from Ewes. J. Dairy Sci..

[63] Quintana Á.R., Perea J.M., Palop M.L., Garzón A., Arias R. (2020). Influence of Environmental and Productive Factors on the Biodiversity of Lactic Acid Bacteria Population from Sheep Milk. Animals.

[64] Quintana Á.R., Perea J.M., García-Béjar B., Jiménez L., Garzón A., Arias R. (2020). Dominant Yeast Community in Raw Sheep’s Milk and Potential Transfers of Yeast Species in Relation to Farming Practices. Animals.

[65] Jiménez L. (2019). Evaluation of the Hygienic-Sanitary and Technological Quality of Manchega Sheep as an Instrument for the Improvement of the Social-Economic and Environmental Viability of the Productive Systems of Milk Sheep. Ph.D. Thesis.

[66] Jiménez Sobrino L., Poveda Colado J.M., Garzón Sigler A.I., Martínez Marín A.L., Núñez Sánchez N., Asensio J.R., Pérez-Guzmán Palomares M.D., Arias Sánchez R. (2018). Composition and Colour Indices of Sheep’s Bulk-Tank Milk Are Influenced by Production Practices. Ital. J. Anim. Sci..

